# The Treatment of Enteric Fever

**Published:** 1892-12-03

**Authors:** 


### ST. MARY'S HOSPITAL.

### The Treatment of Enteric Fever.

The treatment of enteric fever at St. Mary's will be discussed under the following headings: (1) The hygienic and dietetic treatment; (2) the treatment by stimulants and drugs; (3) the treatment of the pyrexia; (4) the treatment of the complications.

1. *The Hygienic and Dietetic Treatment.*—The patient is put to bed in a large and airy ward, the temperature of which is maintained at about 60 deg. F. The bed should be firm, and is so arranged that the patient can be reached from either side. The coverings should be light, but at the same time adequate, to protect the patient from changes of temperature in the ward. The patient is under no circumstances allowed to get out of bed, and the bed pan and urine bottle are used throughout the whole attack, so that the recumbent position is maintained, a point of considerable importance. The stools are examined daily by the house physician or sister of the ward, and are thoroughly disinfected before being thrown away. The patient is sponged twice daily with cool or tepid water, to which vinegar may or may not be added. The temperature of the water may be modified to suit the feelings of the patient. The sponging is carried out more frequently when circumstances, such as restlessness, &c., require it. The temperature is under ordinary circumstances taken every four hours. The diet consists exclusively of liquids, and is given as a rule in the form of milk and beef tea. Two to three pints of milk and one to one and a half pints of beef tea are given in the twenty-four hours, and this amount is rarely exceeded. The milk and beef-tea are given alternately at intervals of two or three hours, according to the discretion of the nurse with regard to disturbing the patient. In severe cases and in emergencies, the patient is fed much more frequently, and the food may have to be given in much more concentrated form. In all cases it is of importance that an exact record should be kept of all foods taken by the patient. The stools are examined daily for curds, and if these are present, some substance such as lime water, carbonate of soda, or seltzer is added to the milk. In many cases it is sufficient to reduce the quantity of milk taken. Should the beef tea excite diarrhœa, as is fairly commonly found to be the case, some substitute such as one of the many preparations of meat extracts or meat jellies may be tried. The patient is allowed to drink freely of cold water, toast water, barley water, or any bland fluid in order to assuage his thirst. Small lumps of ice may be sucked for the same purpose. In those cases in which the milk, given with the precautions above mentioned, cannot be digested, it may be tried in the peptonised form, or Nestle's food may be given. The greatest care is observed during early convalescence with regard to the resumption of solid food. It is usual at St. Mary's to wait at least seven days after the temperature is normal before any attempt is made to give solid food. A little custard pudding is often much appreciated to begin with. At first slops may be given, or an egg beaten up or lightly poached. Then boiled fish may be added to the dietary and then some chicken. Potatoes and other vegetables are better avoided at first. A too early resumption of the upright position is to be avoided, as it is often followed by faintness, or even by syncope.

*The Treatment by Stimulants and Drugs.*—Many of the cases of enteric fever at St. Mary's are treated throughout the whole of the attack without the use of alcoholic stimulants. It is the rule to avoid the giving of stimulants unless they are called for by special indications. The chief factors upon which the administration of alcohol in enteric fever depend are to be found in the age, previous habits, and actual condition of the patient. Young subjects require less alcohol than older ones. Alcohol is generally necessary in the cases of those people who have been of intemperate habits, and must often be given even in an early stage of the disease. The special indications which it is held call for the use of alcohol are as follows: A feeble, frequent, dicrotic pulse, a short and weak first sound of the heart, general muscular weakness, especially if associated with muscular tremors and subsultus tendinum, a dry, glased, and fissured tongue, with sordes on the lips and teeth, and delirium. It is of the utmost importance that the heart should be examined daily, as weakness and shortness of the first sound are often the first indications that alcohol is necessary. Moreover, the effect of the alcohol on the patient should always be carefully observed. In those cases in which the patient is treated by means of tepid baths, alcohol is required both before and after immersion.

It is best given in the form of brandy. The amount given at St. Mary's rarely exceeds twelve ounces in the twenty-four hours, and the quantity prescribed must be administered at regular intervals.

In the absence of complications the employment of general medicinal treatment is usually avoided at St. Mary's. A mixture of one or two grains of the sulphate of quinine, with a small quantity of mineral acid, is sometimes given with advantage. Occasionally, too, antiseptic remedies, such as the liq. hydrarg. perchlor are used, but the question of their usefulness is still undecided. In an early stage of the disease, with considerable general prostration, Dr. Broadbent often prescribes two or three grains of calomel or grey powder with exceedingly beneficial results. It is scarcely necessary to add that this treatment must only be adopted in suitable cases and with extreme care.

In the latter stages of the attack digitalis, ether, and ammonia are often of the greatest service, and especially is this observable in those cases in which heart failure is threatened.

*The Treatment of the Pyrexia.*—If the temperature does not rise above 103 deg. F., it is not considered necessary to reduce it by medicinal means.

Should the temperature rise above 103 deg. F., it is advisable to take measures to reduce it. Dr. Broadbent advocates the use of the graduated bath, but this treatment is rarely adopted at St. Mary's, except in cases of hyperpyrexia.

Should, however, this treatment be deemed necessary, the method of procedure is as follows: Whenever the temperature of the patient exceeds 103 deg. F., he is lifted into a bath, the temperature of which should be about 83 deg. F. The length of immersion is in accordance with the effect produced. The temperature of the bath is gradually reduced to 65 deg. or 70 deg. F. After the temperature of the patient has fallen two degrees, or if shivering be observed, he is replaced in bed, dried, and covered up. It is usual to administer a little brandy before and after the immersion. In order that the best results may be obtained from this treatment it must be begun early. Baths are absolutely contra-indicated sbould bæmorrhage or peritonitis supervene.

Other methods of reducing the temperature are by cold or tepid sponging, and by the use of quinine in the form of the sulphate or hydrobromate, in doses ranging from five to twenty grains. Salicylates are seldom used at St. Mary's on account of their depressing effect, and antipyrin practically never. Antifibrin is also rarely employed.

When quinine is given in large doses, which are generally necessary to produce a definite fall of temperature of any duration, it is best administered with opium, which obviates the sickness which is produced by large doses of the former drug.

Treatment of the Complications.

*Diarrhœa and Constipation.*—If the number of stools does not exceed three in the twenty-four hours it is not the custom to check the diarrhœa. Should they exceed this number, an enema of starch and opium may be administered. The injection usually consists of twenty to thirty drops of the tincture of opium in two ounces of starch. This may be repeated when necessary. If, however, it fails to check the diarrhœa, opium or some intestinal astringent, such as sulphuric acid, logwood, &c., may be administered by the mouth. If, on the other hand, there is constipation, small doses (ʒi.—iii.) of castor oil may be given up to the end of the first week. After that constipation is best relieved by means of a small enema of soap and water. An enema of glycerine (ʒi.—ii.) is sometimes very efficacious, but in some cases it has been supposed to excite peristalsis of the bowel above the rectum, and thus to be a source of danger. The constipation that sometimes ensues during convalescence is best treated with castor oil.

*Hæmorrhage* is treated with full doses of opium, *i.e.*, ʒss.—ʒj. of the tincture. An ice pad may also be placed over the calcum. Ergot or turpentine may sometimes be useful. It is better to avoid feeding the patient by the mouth for twenty-four hours after hæmorrhage has occurred.

For *Peritonitis and Perforation* opium, in large doses, is the only treatment that is of any service.

For *Tympanitis* opium again is the best remedy, and is most advantageously given in the form of a pill. Ice compresses to the surface of the abdomen may also be used. A turpentine enema, or the passage of the long rectal tube is sometimes successful.

*Pulmonary Complications* call for stimulants. Gentle local counter irritation is also of service.

*Sleeplessness* is treated by opium, if cold or tepid sponging fail to relieve it. Sulphonal is often temporarily successful. If sleeplessness occurs without any obvious cause, it is advisable to examine the bladder, and, if found to be full, a catheter must be passed and the urine drawn off.

*Vomiting* is treated by restricting the diet and by the sub-cutaneous injection of morphia. The patient may, if necessary, be temporarily fed by enemata.

Other complications must be treated on general principles. It is of the utmost importance that the patient is kept scrupulously clean; and, should bed sores threaten, the part is sponged with a spirit lotion and the patient placed on a water bed.

